# Graphene’s nonlinear-optical physics revealed through exponentially growing self-phase modulation

**DOI:** 10.1038/s41467-018-05081-z

**Published:** 2018-07-11

**Authors:** Nathalie Vermeulen, David Castelló-Lurbe, Mulham Khoder, Iwona Pasternak, Aleksandra Krajewska, Tymoteusz Ciuk, Wlodek Strupinski, JinLuo Cheng, Hugo Thienpont, Jürgen Van Erps

**Affiliations:** 1 Brussels Photonics, Dept. of Applied Physics and Photonics, Vrije Universiteit Brussel, Pleinlaan 2, 1050 Brussel, Belgium; 2 Institut Universitari de Ciències dels Materials, Universitat de València, Catedrático Agustín Escardino 9, 46980 Paterna, Spain; 3 Institute of Electronic Materials Technology, Wolczynska 133, 01-919 Warsaw, Poland; 4 Faculty of Physics, Warsaw University of Technology, Koszykowa 75, 00-662 Warsaw, Poland; 5 The Guo China-US Photonics Laboratory, Changchun Institute of Optics, Fine Mechanics and Physics, Chinese Academy of Sciences, 3888 Eastern South Lake Road, 130033 Changchun, Jilin China

## Abstract

Graphene is considered a record-performance nonlinear-optical material on the basis of numerous experiments. The observed strong nonlinear response ascribed to the refractive part of graphene’s electronic third-order susceptibility *χ*^(3)^ cannot, however, be explained using the relatively modest *χ*^(3)^ value theoretically predicted for the 2D material. Here we solve this long-standing paradox and demonstrate that, rather than *χ*^(3)^-based refraction, a complex phenomenon which we call saturable photoexcited-carrier refraction is at the heart of nonlinear-optical interactions in graphene such as self-phase modulation. Saturable photoexcited-carrier refraction is found to enable self-phase modulation of picosecond optical pulses with exponential-like bandwidth growth along graphene-covered waveguides. Our theory allows explanation of these extraordinary experimental results both qualitatively and quantitatively. It also supports the graphene nonlinearities measured in previous self-phase modulation and self-(de)focusing (*Z*-scan) experiments. This work signifies a paradigm shift in the understanding of 2D-material nonlinearities and finally enables their full exploitation in next-generation nonlinear-optical devices.

## Abstract (web summary)

Graphene enables extraordinary nonlinear-optical refraction, far exceeding predictions based on conventional nonlinear-susceptibility theory. Here, Vermeulen et al. show that rather than the nonlinear susceptibility, a complex saturable refraction process is central to graphene’s unusual behavior.

## Introduction

Over the past several years numerous experiments have been carried out to study the response of graphene in parametric nonlinear-optical processes like four-wave mixing^1–5^, third-harmonic generation^6,7^, self-(de)focusing^8–12^, and self-phase modulation (SPM)^13–15^. These measurements mostly pointed at an extremely high effective third-order susceptibility $\left| {\chi _{{\mathrm{eff}}}^{\left( 3 \right)}} \right| \propto 10^{ - 7}\,{\mathrm{esu}}$ in the 2D material, much higher than what theory predicts for graphene’s electronic third-order susceptibility $\left| {\chi _{{\mathrm{th}}}^{\left( 3 \right)}} \right| \propto 10^{ - 9}\,{\mathrm{esu}}$^16–19^ in the density-matrix framework at the single-particle level^20,21^. The origin of this large discrepancy between theory and experiments has so far remained unknown, hindering the full exploitation of graphene in nonlinear-optical applications. Several recent experiments also revealed that the sign of graphene’s effective $\chi _{{\mathrm{eff}}}^{(3)}$ is negative^10,11,14^, but again without conclusive theoretical support. Hence, both the negative sign of the experimentally observed nonlinearity and its large quantitative discrepancy as compared to the theoretically predicted response have unclear origins at this point. This holds not only for graphene experiments with free-space optical excitation^1,6–13^ but also for the cases where graphene on a photonic chip is examined using waveguided excitation beams^2–4,13,14^. The latter configuration is particularly interesting from a practical point-of-view as it allows exploring the potential of graphene for next-generation on-chip nonlinear-optical devices (e.g., record-performance on-chip frequency-comb and supercontinuum light sources) opening up new applications in optical telecommunication, biomedical imaging, absorption spectroscopy, and many other domains^22–26^.

To investigate graphene’s intrinsic nonlinear physics, one should ideally be working with a substrate free from any nonlinear-optical effects that could interact with those of the 2D material. In case of graphene-covered photonic chips, the core of the on-chip waveguides should preferably be made of SiO_2_ for the larger part, since the nonlinearity of SiO_2_ is orders of magnitude smaller than that of other materials typically employed for photonic chip fabrication (Si, InP, Si_3_N_4_,…)^21,27^ and because its dielectric nature provides electrical isolation of the graphene.

In this paper, we solve the long-standing theory-versus-experiments paradox in graphene nonlinear optics by investigating SPM of optical pulses in graphene-covered SiO_2_-core waveguides. We observe an extraordinary spectral broadening behavior, and demonstrate through an in-depth theoretical and experimental analysis that the underlying nonlinear physics does not depend on refraction induced by graphene’s electronic *χ*^(3)^, but instead on a much more intricate phenonemon that we refer to as saturable photoexcited-carrier refraction (SPCR).

## Results

### Spectral broadening in graphene-covered SiO_2_-core waveguides

**Fig. 1:**
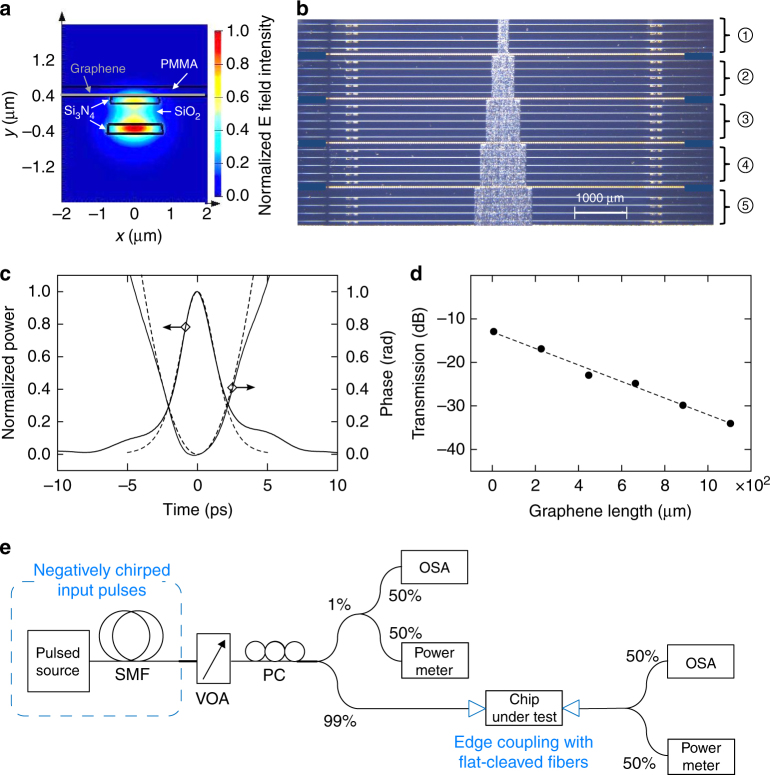
Illustration of the setup used for the experiments and its constituents. **a** Cross-section of the SiO_2_-core waveguides covered by graphene with poly(methyl methacrylate) (PMMA), and numerically simulated distribution of the electric (*E*) field intensity. **b** Top-view microscope image of the SiO_2_-core waveguides with regions 1–5 covered by, respectively, 220, 440, 660, 880, and 1100 μm-long graphene sections with PMMA (white areas in center). **c** Temporal power profile and phase profile of the negatively chirped input pulse measured with the frequency-resolved optical gating (FROG) technique (solid curves) and the corresponding fit assuming a quadratic phase profile with *C*_0_ = −0.2 and *T*_FWHM,0_ = 3 ps (dashed curves). **d** Waveguide transmission as a function of graphene length as measured (dots) and the corresponding linear fit with slope *α*_eff_ = 4605 m^−1^ (dashed curve). **e** Setup for the transmission and spectral broadening measurements. SMF single-mode fiber, VOA variable optical attenuator, PC polarization controller, OSA optical spectrum analyzer

The graphene-covered SiO_2_-core waveguides employed in our experiments, here also referred to as hybrid waveguides, feature a cross-section as illustrated in Fig. 1a. The graphene top layer grown by means of chemical vapor deposition features a carrier density of +6.5 × 10^12^ cm^−2^ as a result of unintentional doping^28^, and is patterned with plasma etching^29^ to create sections with variable length *L* between 220 and 1100 μm on the waveguides as shown in Fig. 1b. More detailed information on the geometrical properties of the waveguides and the material characteristics of the graphene top layer can be found in Methods, Supplementary Notes 1–2 and Supplementary Figures 1–3. The waveguides’ fundamental quasi-transverse electric (TE) mode is excited with picosecond (ps)-duration input pulses at *λ* = 1563 nm in the telecom band (see also Methods). The input can be described by a pulse amplitude $A\left( {0,t} \right) = \left| {A\left( {0,t} \right)} \right|{\mathrm{exp}}\left[ {{\mathrm{i}}\varphi (0,t)} \right] = \sqrt {P_0} U\left( {0,t} \right){\mathrm{exp}}\left[ { - {\mathrm{i}}C_0t^2/T_0^2} \right]$ featuring a modest incoupled peak power *P*_0_ up to 2.7 W, a temporal shape *U*(0, *t*) = sech(*t*/*T*_0_), a full-width-at-half-maximum pulse duration *T*_FWHM,0_ = 1.76 *T*_0_ = 3 ps (Fig. 1c) and a quasi-linear input chirp $\partial _t\varphi \left( {0,t} \right) = \left( { - 2C_0t} \right)/T_0^2$ with a negative chirp parameter *C*_0_ = −0.2 (Fig. 1c). We work with negatively chirped input pulses as these allow the graphene to induce the strongest spectral broadening of the pulses in the low-power regime^14^. The resulting broadening factor is defined as the square of the pulses’ rms spectral width^30^ at the waveguide output facet (*z* = *z*_out_) divided by the corresponding value at the input (*z* = 0), i.e., *μ*_2_(*z*_out_)/*μ*_2_(0) with $\mu _2(z) = \left( {{\int}_{ - \infty }^\infty \left| {\partial _tA} \right|^2{\mathrm{d}}t} \right)/\left( {{\int}_{ - \infty }^\infty \left| A \right|^2{\mathrm{d}}t} \right)$. Prior to the broadening experiments, we measure the transmission of the pulses in the hybrid waveguides to characterize the graphene-induced optical absorption as a function of graphene length *L*. We observe a linearly increasing effective loss along graphene length with a slope *α*_eff_ = 4605 m^−1^ (Fig. 1d). The general experimental setup used for both the transmission and spectral broadening measurements is illustrated in Fig. 1e.

**Fig. 2:**
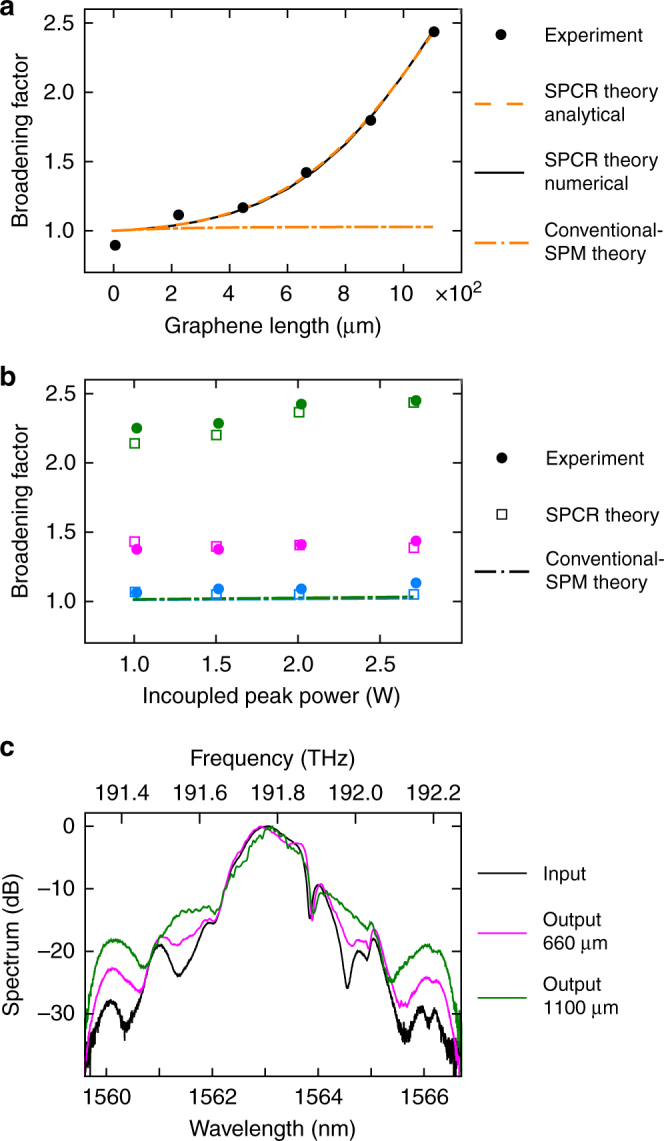
Results of the broadening experiments. **a** Broadening factor as a function of graphene length for an incoupled peak power of 2.7 W. **b** Broadening factor as a function of incoupled peak power indicated in blue, magenta, and green for graphene lengths of 220, 660, and 1100 μm, respectively. Both graphs show the experimental data points (with a measurement error below 5 %), the results from our saturable photoexcited-carrier refraction (SPCR) theory (numerical solution from Eqs. (4) and (5), and analytical solution from Eq. (6)), and the results from the theory for conventional self-phase modulation (SPM) using graphene’s strong effective *χ*_eff_^(3)^ = −10^−7^ esu as extracted from earlier experiments. **c** Normalized experimental input spectrum and output spectra for graphene lengths of 660 and 1100 μm excited with an incoupled peak power of 2.7 W. The spectra are normalized to their respective peak power values

When carrying out the spectral broadening experiments, we observe, as shown in Fig. 2a–c, an extraordinary exponential-like growth of the graphene-induced broadening as a function of its length up to at least *L* = 1100 μm, while its dependence on input power is very weak. In the next sections, we analyze this remarkable behavior to reveal the nonlinear physics taking place in the 2D material.

### Comparison with conventional SPM

It is widely accepted that spectral broadening of optical pulses in a nonlinear material typically originates from SPM^31^ based on the material’s electronic third-order susceptibility *χ*^(3)^. This process relies on the *χ*^(3)^-induced nonlinear part of the refractive index ${\mathrm{\Delta }}n_{^{\left( {\mathrm{3}} \right)}}\left( {z,t} \right) \propto \chi ^{(3)}\left| {A\left( {z,t} \right)} \right|^2$, which impacts the pulse chirp (and hence the spectral width) through the chirp-index relation $\partial _t\varphi \left( {z,t} \right) \propto \partial _t{\int}_0^z {\mathrm{\Delta }}n\left( {z\prime ,t} \right){\mathrm{d}}z\prime$. As such, the chirp induced by conventional *χ*^(3)^-based SPM obeys the following well-established expression^31^ (after introducing *τ* = *t*/*T*_0_ and $\left| {\tilde U\left( \tau \right)} \right|^2 = \left| {U\left( {0,t} \right)} \right|^2$):

1 $$\partial _\tau \varphi \left( {z,\tau } \right) = K\,P_0\,\partial _\tau \left| {\tilde U} \right|^2\left. {\left[ {\Theta _1{\kern 1pt} {\mathrm{e}}^{ - \alpha _{{\mathrm{eff}}}z}{\kern 1pt} \alpha _{{\mathrm{eff}}}^{ - 1}} \right]} \right|_z^0$$

with the proportionality constant *K* = *γ* = *f*(*χ*^(3)^) being the medium’s nonlinear parameter *γ* function of *χ*^(3)^ (ref.^31,32^) or function of weighted *χ*^(3)^ contributions in case of hybrid waveguides^33^, with $\left. {\left[ {\Theta _1{\kern 1pt} {\mathrm{e}}^{ - \alpha _{{\mathrm{eff}}}z}{\kern 1pt} \alpha _{{\mathrm{eff}}}^{ - 1}} \right]} \right|_z^0$ representing the effective length, and with *Θ*_1_ = 1 (the latter function is introduced to facilitate comparison with other chirp formulas further on). The resulting spectral width thus varies linearly with input power *P*_0_ and with effective length^31^, leveling off starting from $z > \alpha _{{\mathrm{eff}}}^{ - 1}$.

Such dependences on power and length, however, are totally different from those seen in our experiments: indeed, as shown by the dash-dot lines in Fig. 2a, b, conventional-SPM theory cannot provide a qualitative nor quantitative description for the observed exponential-like growth of the broadening all the way to *L* = 1100 μm (i.e., far beyond $z = \alpha _{{\mathrm{eff}}}^{ - 1} = 217\,{\mathrm{\mu m}}$), and likewise it cannot provide an adequate description for the measured broadening as a function of power (note that a zoom-in of the conventional-SPM modeling curves is provided in Supplementary Figure 4 and discussed in Supplementary Note 3).

### Confirmation of chirping-based nature

As the observed spectral broadening in the graphene-covered waveguides cannot be explained quantitatively nor qualitatively by conventional *χ*^(3)^-dependent SPM, we have to reconsider the very basics of the process at work and verify whether it is effectively based on chirping or rather on absorption-induced pulse shaping.

If absorption-induced pulse shaping governed the spectral broadening in the graphene-covered waveguides, then the phase *φ*(*z*, *t*) of the pulse envelope would not change along the propagation path, and when injecting different pulses that share the same input amplitude |*A*(0, *t*)|, the evolution in |*A*(*z*, *t*)| along the propagation path would be the same as well for these pulses. In that case, when performing broadening experiments for two different input pulses p1 and p2 with opposite input chirp parameters $C_0^{{\mathrm{(p1)}}} \approx - C_0^{{\mathrm{(p2)}}}$ but with the same input amplitude |*A*(0, *t*)| (and hence the same *P*_0_ and *T*_FWHM,0_), the resulting spectral widths $\mu _2^{{\mathrm{(p1)}},{\mathrm{(p2)}}}\left( z \right)$ along the waveguides would obey the following relation:

2 $$	\mu _2^{{\mathrm{(p1)}}}\left( z \right) - \mu _2^{{\mathrm{(p2)}}}\left( z \right) \\ 	 =\frac{{4\left( {\left( {C_0^{{\mathrm{(p1)}}}} \right)^2 - \left( {C_0^{{\mathrm{(p2)}}}} \right)^2} \right){\int}_{ - \infty }^\infty \left( {t^2/T_0^4} \right)\left| {A^{{\mathrm{(p1)}}}\left( {z,t} \right)} \right|^2{\mathrm{d}}t}}{{{\int}_{ - \infty }^\infty \left| {A^{{\mathrm{(p1)}}}\left( {z,t} \right)} \right|^2{\mathrm{d}}t}} \approx 0$$

for all values of *z*. Dividing Eq. (2) by the input spectral width $\mu _2^{{\mathrm{(p1)}}}\left( 0 \right) \approx \mu _2^{{\mathrm{(p2)}}}\left( 0 \right) \equiv \mu _{2,{\mathrm{in}}}$ yields the following relation between the broadening factors $\mu _2^{{\mathrm{(p1)}},{\mathrm{(p2)}}}\left( z \right)/\mu _{2,{\mathrm{in}}}$ for input pulses p1 and p2:

3 $$\mu _2^{{\mathrm{(p1)}}}\left( z \right)/\mu _{2,{\mathrm{in}}} \approx \mu _2^{{\mathrm{(p2)}}}\left( z \right)/\mu _{2,{\mathrm{in}}}$$

for all values of *z*. Logic tells us then that if in reality the relation of Eq. (3) is not fulfilled for all values of *z*, then the underlying broadening mechanism cannot rely on absorption-induced pulse shaping and instead has to be based on chirping.

**Fig. 3:**
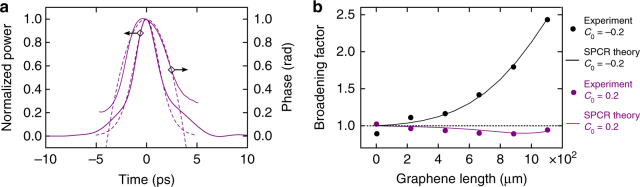
Characteristics of positively chirped input pulses and corresponding broadening results. **a** Temporal power profile and phase profile of the positively chirped input pulse p2 measured with the frequency-resolved optical gating (FROG) technique (solid curves) and the corresponding fit assuming a quadratic phase profile with $C_0^{{\mathrm{(p2)}}} = 0.2$ and $T_{{\mathrm{FWHM}},0}^{{\mathrm{(p2)}}} = 3$ ps (dashed curves). **b** Broadening factor for input pulse p2 with $C_0^{{\mathrm{(p2)}}} = 0.2$ as a function of graphene length (in purple). Both the experimental data points and the results from our saturable photoexcited-carrier refraction (SPCR) theory are shown. For comparison we also plot again the broadening factors for input pulse p1 with $C_0^{{\mathrm{(p1)}}} = - 0.2$ (in black) as shown in Fig. 2a

With this theory in mind, and taking the input pulse used for Fig. 2a as pulse p1 with *P*_0_ = 2.7 W, *T*_FWHM,0_ = 3 ps, and $C_0^{{\mathrm{(p1)}}} = - 0.2$, we subsequently generate a second input pulse p2 with the same peak power and pulse duration and with $C_0^{{\mathrm{(p2)}}} = + 0.2$ (Fig. 3a). Hereto, we make use of our pulse-chirp control method which is explained in Methods. Using this newly generated input pulse p2, we again perform spectral broadening measurements with the graphene-covered waveguides, yielding the broadening factors shown in purple in Fig. 3b. There is a very large difference between the broadening data for input pulse p1 shown in black (cf. Fig. 2a) and those for input pulse p2: whereas the former exhibit strong spectral broadening, the latter show spectral narrowing (i.e., broadening factors smaller than 1). In other words, Eq. (3) clearly is not fulfilled, so the dominant broadening process in the graphene-covered waveguides cannot rely on absorption-induced pulse shaping and instead must be based on chirping. In addition, this observation of spectral narrowing for a positively chirped input pulse points at a self-defocusing nonlinearity being at play here. In what follows, we investigate if the underlying physics can be explained by chirping effects induced by photoexcited free carriers in the graphene.

### Analysis based on SPCR

While the optical pulses are traveling through the graphene-covered waveguides, they experience graphene-induced optical absorption and as such give rise to photoexcited free carriers with a density *N*_c_(*z*, *t*), altering the refractive index^34^ Δ*n*_carriers_ ∝ *N*_c_(*z*, *t*) and yielding pulse chirping $\partial _t\varphi \left( {z,t} \right) \propto \partial _t{\int}_0^z {\mathrm{\Delta }}n_{{\mathrm{carriers}}}\left( {z\prime ,t} \right){\mathrm{d}}z\prime \propto \partial _t{\int}_0^z N_{\mathrm{c}}\left( {z\prime ,t} \right){\mathrm{d}}z\prime$. This phenomenon is generally known as free-carrier-refraction (FCR)-induced chirping and has already been studied in waveguides made of 3D-semiconductor materials^34–38^. Yet, in the specific case of graphene-covered waveguides, FCR-induced chirping turns out to be much more complex than in the 3D-semiconductor-based waveguides where it has been examined so far. The reason is that here the time evolution of *N*_c_ along the pulse propagation path strongly depends on the possible occurrence of saturation (i.e., *N*_c_ ~ *N*_sat_) in the graphene due to Pauli blocking^39^, and therefore we refer to the resulting chirping as ‘saturable photoexcited-carrier refraction (SPCR)’-induced chirping. To model this new phenomenon, we employ the following phenomenological equations for the propagation of the optical pulses in the hybrid waveguides and for the time evolution of the free-carrier density:

4 $$\partial _zA\left( {z,t} \right) = - \frac{{\alpha _{{\mathrm{eff}}}}}{2}A\left( {z,t} \right) - {\mathrm{i}}\sigma _{{\mathrm{FCR}}}N_{\mathrm{c}}^{{\mathrm{gr}}}\left( {z,t} \right)A\left( {z,t} \right)$$

5 $$\partial _tN_{\mathrm{c}}^{{\mathrm{gr}} + {\mathrm{wg}}}\left( {z,t} \right) =	 \frac{{\eta _{{\mathrm{1PA}}}}}{{\hbar \omega }}\left[ {1 - \frac{{N_{\mathrm{c}}^{{\mathrm{gr}} + {\mathrm{wg}}}\left( {z,t} \right)}}{{N_{{\mathrm{sat}}}}}} \right]\left| {A\left( {z,t} \right)} \right|^2 \\ 	- \frac{{N_{\mathrm{c}}^{{\mathrm{gr}} + {\mathrm{wg}}}\left( {z,t} \right)}}{{\tau _{\mathrm{c}}}}$$

In Eq. (4), *σ*_FCR_ represents the graphene-induced FCR coefficient, and $N_{\mathrm{c}}^{{\mathrm{gr}}}$ the photoexcited free-carrier density in graphene. We note that there is no explicit free-carrier-induced absorption term included in the phenomenological model in view of the linear absorption trend along graphene length shown in the transmission measurements of Fig. 1d. This absorption trend is entirely modeled using the constant coefficient *α*_eff_. In Eq. (5), $N_{\mathrm{c}}^{{\mathrm{gr}} + {\mathrm{wg}}}$ is the photoexcited free-carrier density over all parts of the hybrid waveguide cross-section where the carriers can exist, *τ*_c_ indicates the effective carrier lifetime determined by relaxation in all aforementioned parts of the waveguide cross-section, *η*_1PA_ is graphene’s efficiency of one-photon-absorption-induced carrier generation, and *ω* = 2*c*/*λ*. We have taken a negative sign for the factor $- {\mathrm{i}}\sigma _{{\mathrm{FCR}}}N_{\mathrm{c}}^{{\mathrm{gr}}}$ in Eq. (4) in view of the nonlinearity’s defocusing nature as phenomenologically observed above, and we have also verified from a physics point of view that for graphene and the free-carrier densities present here the nonlinearity sign is indeed negative.

**Fig. 4:**
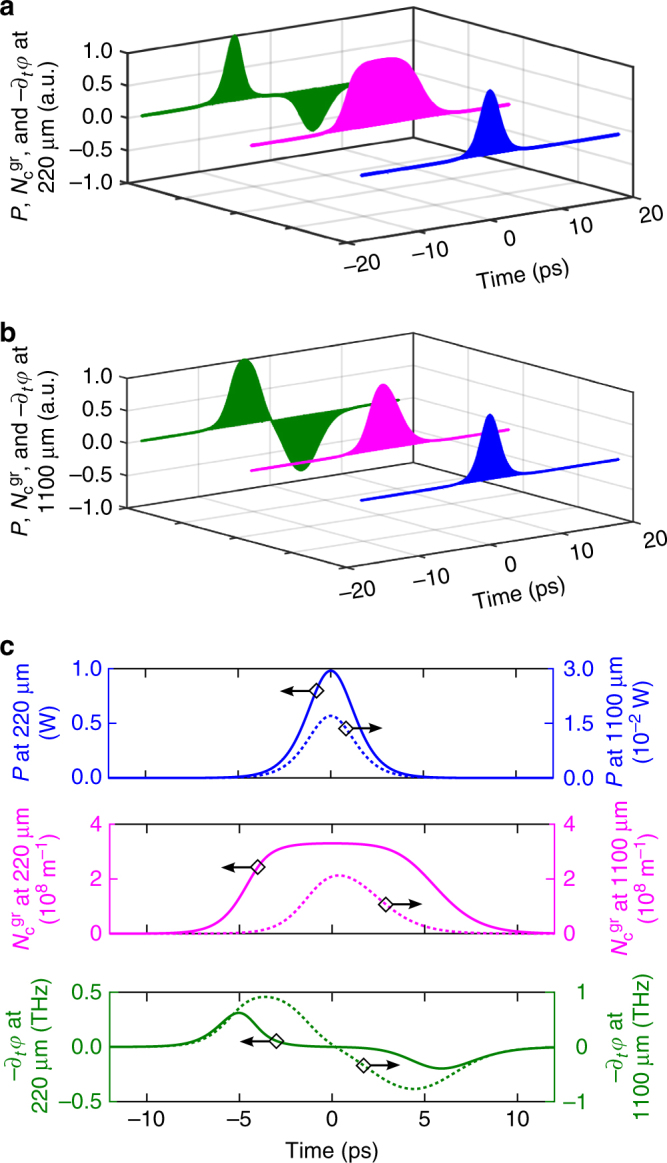
Time evolution of the optical and carrier-related quantities at different distances in the graphene-covered SiO_2_-core waveguides: 3D illustration of the time evolution of the optical power *P* (blue), the graphene-generated free-carrier density $N_{\mathrm{c}}^{{\mathrm{gr}}}$ (magenta), and the instantaneous frequency −∂_*t*_ *φ* (green) at **a** 220 μm and **b** 1100 μm propagation distance. Both 3D graphs show normalized quantities. **c** Time evolution of the same quantities, but with the numerically obtained absolute values displayed and with the solid and dashed curves corresponding to, respectively, 220 and 1100 μm propagation distance

In the cross-section of the electrically isolating SiO_2_-core waveguides covered with graphene, the photoexcited carriers can only exist in the graphene top layer. Hence, $N_{\mathrm{c}}^{{\mathrm{gr}} + {\mathrm{wg}}}$ in Eq. (5) becomes $N_{\mathrm{c}}^{{\mathrm{gr}}}$ as used in Eq. (4), and *η*_1PA_ = *α*_eff_. We can readily quantify the different parameter values in Eqs. (4) and (5) since they have been measured either in the experiments presented here or in previous works (Table 1). Particularly important is the value of *τ*_c_, which is known to be around 1 ps for photoexcited carriers in graphene on top of a dielectric^39,40^ and thus close to *T*_0_ of the input pulses used. We note that the values of *τ*_c_ and *N*_sat_ in Table 1 were adopted from ref.^39^, where graphene was investigated deposited on a dielectric substrate and excited with ps-scale telecom wavelength pulses similarly as in our experiments. *τ*_c_ is defined as an effective decay time providing a phenomenological description for the combined impact of the different relaxation mechanisms taking place in the graphene^39,41^. As shown in Fig. 2a, b (and also in Fig. 3b), we obtain an excellent qualitative and quantitative correspondence between our SPCR theory modeled by Eqs. (4) and (5) and our experimental results. We remark that our SPCR theory can adequately describe the observed broadening behavior also in case the actual values for, e.g., *τ*_c_ and *N*_sat_ in our graphene-covered SiO_2_-core waveguides would somewhat deviate from those in Table 1 (see Supplementary Note 5 and Supplementary Figure 6). Fig. 4 illustrates the origin of the extraordinary exponential-like growth in Fig. 2a of the broadening factor over distance in the graphene-covered SiO_2_-core waveguides. In Fig. 4a, we see that at short propagation distances the free-carrier density in the graphene has a temporal profile that rises together with the leading edge of the optical pulse, but saturates in the high-power pulse center (i.e., *N*_c_ ~ *N*_sat_). As such, $\partial _t\varphi \left( {z,t} \right) \propto \partial _t{\int}_0^z N_{\mathrm{c}}\left( {z\prime ,t} \right){\mathrm{d}}z\prime$ is negligible around the pulse center so that we obtain a peculiar chirping profile with its extrema situated in the low-power pulse tails, and hence the spectral broadening is considerable but not yet strong. In contrast, after propagating over longer graphene-covered waveguide distances (Fig. 4b), the pulse contains significantly less power due to the graphene absorption (Fig. 4c), and saturation no longer occurs. In this case, there will be strong, maximal chirping in the vicinity of the high-power pulse center, yielding much more efficient broadening and as such an exponential-like increase of the spectral bandwidth over graphene length. We point out that the described chirping behavior is unique as it establishes both up-chirping and down-chirping along variable chirp profiles. This is in contrast to FCR in 3D-semiconductor waveguides yielding only up-chirping or blue-shifting of the pulses^35–37^, and also opposite to conventional SPM featuring only a fixed-shaped chirp profile^31^. The determining factors for this novel behavior are the short *τ*_c_ for photoexcited carriers in graphene and the saturability of the 2D material.

**Table 1:** Values of the parameters in Eqs. (4–6) for graphene-covered SiO_2_-core waveguides

	Value	Reference
*α*_eff_ (m^−1^)	4605	Transmission exp.
*τ*_c_ (ps)	1	39
*N*_sat_ (m^−1^)^a^	$\sqrt {10^{17}}$	39
*σ*_FCR_ (−)	1(±0.2) × 10^−5^	Broadening exp.^b^

^a^$N_{\mathrm{c}}^{{\mathrm{gr}}}$ and *N*_sat_ are 1D graphene carrier densities within the waveguide cross-section

^b^The uncertainty interval on the FCR coefficient is extracted from the data measured at different power levels

The unusually weak power dependence seen in Fig. 2b can be understood from the following analytical approximation for the SPCR-induced pulse chirp in our experiments with *τ*_c_ ≈ *T*_0_:

6 $$\partial _\tau \varphi \left( {z,\tau } \right) = \left[ { - \sigma _{{\mathrm{FCR}}}\frac{{\alpha _{{\mathrm{eff}}}\tau _{\mathrm{c}}}}{{\hbar \omega }}} \right]P_0\,\partial _\tau \left| {\tilde U} \right|^2\left. {\left[ {\Theta _2{\mathrm{e}}^{ - \alpha _{{\mathrm{eff}}}z}\,\alpha _{{\mathrm{eff}}}^{ - 1}} \right]} \right|_z^0$$

with *Θ*_2_ = 1/(1 + *x*), where $x = \left( {\eta _{{\mathrm{1PA}}}{\kern 1pt} \tau _{\mathrm{c}}{\kern 1pt} {\mathrm{e}}^{ - \alpha _{{\mathrm{eff}}}z}{\kern 1pt} P_0\left| {\tilde U} \right|^2} \right)/\left( {\hbar \omega N_{{\mathrm{sat}}}} \right)$. As opposed to the chirp for conventional *χ*^(3)^-based SPM (Eq. (1)), the chirp of Eq. (6) does not simply scale linearly with *P*_0_ in the numerator, but instead exhibits a reduced dependence on the power due to the presence of *P*_0_ in the denominator of *Θ*_2_. This weak power dependence implies that strong broadening with an exponential-like growth over distance can be obtained even at very low input powers, and at the same time shows that the experimental results presented here cannot be ascribed to modulational-instability-induced spectral changes with power-dependent gain. It should be noted that, whereas the relative powers in the spectral sidebands of Fig. 2c increase when going from short to long graphene lengths, their absolute powers decrease because of the strong graphene-induced one-photon absorption which occurs at all wavelengths in the spectra. Still, there will be potential for actually creating new wavelengths at weak pump powers when further optimizing the balance between the one-photon absorption efficiency and the SPCR efficiency, so that the spectral sidebands could grow also in absolute power. Possible approaches for optimizing this balance are, e.g., by varying graphene’s doping level or by changing the pump wavelength.

Finally, comparing Eq. (6) with Eq. (1) also shows that the quantity −*σ*_FCR_ *α*_eff_ *τ*_c_ /(ℏ*ω*) takes up the role of proportionality constant *K*. The most striking features of this quantity are its negative sign and its dependence on graphene’s FCR efficiency rather than on the electronic *χ*^(3)^.

### Pertinence to other on-chip SPM measurements

In previous work^14^, we investigated spectral broadening in graphene-covered semiconductor (namely silicon (Si)) waveguides and excited with negatively chirped ps pulses that were quasi-identical to those used here. The graphene-induced broadening in graphene-on-Si presented there shows a very different tendency as compared to that seen here for graphene-on-SiO_2_: it exhibits a linear effective-length dependence in line with the conventional-SPM model of Eq. (1) characterized by *K* = *γ*_gr-on-Si_ = −1.7 × 10^3^ W^−1^ m^−1^, yielding $\chi _{{\mathrm{eff}}}^{(3)} = - 10^{ - 7}$ esu for the graphene top layer. Furthermore, the broadening measured there in graphene-on-Si is of the order of a few percent, which is small compared to the broadening well in excess of 200% observed here in graphene-on-SiO_2_ (Fig. 2a, b).

**Fig. 5:**
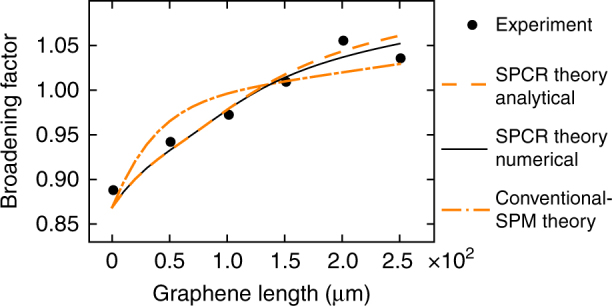
Broadening factor as a function of graphene length in graphene-covered Si waveguides excited with an incoupled peak power of 1.7 W. The graph shows the experimental data points (data taken from ref. ^14^), the results from our saturable photoexcited-carrier refraction (SPCR) theory (numerical and analytical solutions from Eqs. (4) and (5)), and the results from the theory for conventional self-phase modulation (SPM) using graphene’s effectively observed *χ*_eff_^(3)^ = −10^−7^ esu. The graphene lengths used here are relatively short because of the strong graphene-induced absorption *α*_eff_ = 30,394 m^−1^ in this waveguide configuration

Although the broadening in graphene-on-Si is in line with the conventional-SPM model, the actual physical process taking place in the graphene is again SPCR and can be described by the model of Eqs. (4) and (5). It should be noted that here the graphene-generated free carriers can also exist in the underlying Si semiconductor waveguide, turning $N_{\mathrm{c}}^{{\mathrm{gr}} + {\mathrm{wg}}}$ in Eq. (5) into the average carrier density defined over the entire cross-section of the hybrid waveguide and *η*_1PA_ = *α*_eff_/*D* with *D* the waveguide thickness (220 nm in this case). The use of spatially averaged carrier densities stems from the often employed semiconductor-based waveguide description as outlined in ref.^32^, and also implies implementing an average decay time value incorporating the decay times of the different constituents and mechanisms in the waveguide cross-section. Due to the nanosecond-scale carrier lifetime in the underlying Si waveguide^32,42^, we here have a much larger decay time^2^ *τ*_c_ than when the carriers remain confined to the graphene (see above where a decay time for only the graphene top layer and not for the electrically isolating SiO_2_-core waveguides could be defined). As shown in the Supplementary Table 1, we can readily quantify the other parameter values for graphene-on-Si in concert with our previous results. When implementing these in Eqs. (4) and (5) to model SPCR in the graphene top layer, we find an excellent qualitative and quantitative agreement with the experimental data for graphene-on-Si, as depicted in Fig. 5. The agreement is in fact significantly better than that obtained for the conventional-SPM model, which confirms that also in our previously reported measurements with graphene-on-Si, SPCR is at the heart of the observed spectral broadening.

By deriving from Eqs. (4) and (5) an approximated analytical solution for the chirp induced in graphene-on-Si with $\tau _c \gg T_0$ (Supplementary Equation 1), we find that here the quantity $- \sigma _{{\mathrm{FCR}}}\sqrt {\left( {\eta _{{\mathrm{1PA}}}{\kern 1pt} T_0} \right)/\left( {\hbar \omega \,P_0} \right)}$ acts as proportionality constant *K*. This quantity produces both the negative sign and the order of magnitude for the extracted effective *γ*_gr-on-Si_. At the same time, it is one order of magnitude smaller than the quantity *K* = −*σ*_FCR_ *α*_eff_ *τ*_c_ /(ℏ*ω*) for the graphene-covered SiO_2_ waveguides (Eq. (6)). This explains why the spectral broadening we observed in the hybrid Si waveguides as shown in Fig. 5 is considerably smaller than that of their SiO_2_ counterparts discussed above. Further information on the SPCR modeling results for the hybrid Si waveguides can be found in Supplementary Note 4 and Supplementary Figure 5.

### Pertinence to free-space *Z*-scan measurements

The impact of our SPCR analysis goes beyond the process of spectral broadening in graphene-covered waveguides: it actually shows that graphene’s nonlinear phase modulation ability measured in any kind of free-space or waveguide-based experiment depends on its free-carrier generation and refraction efficiencies, rather than on its electronic *χ*^(3)^. Sophisticated free-space *Z*-scan techniques were developed over the years for distinguishing the electronic *χ*^(3)^ contribution from two-photon-absorption-generated free-carrier contributions in 3D semiconductors^34,43–45^; their application to graphene with one-photon-absorption-induced carriers is yet to be explored.

The currently available standard *Z*-scan measurement results (or *Z*-scan-like phase modulation results) for graphene on dielectric substrates and excited with ~100 fs pulses can be fitted using the macroscopic formula Δ*n* ∝ *χ*_eff_^(3)^|*A*|^2^ along the conventional-SPM notation^9–12^. In these experiments *τ*_c_(∝ps) $\gg T_0$, and as shown above, the underlying SPCR physics indeed produces conventional-SPM-like behavior for this lifetime/pulse-length ratio. The $\chi _{{\mathrm{eff}}}^{(3)}$ extracted from these *Z*-scan experiments consistently is of the order of 10^−7^ esu^9–12^.

In contrast, *Z*-scan results obtained with longer, ps-scale pulse lengths *T*_0_ ≈ *τ*_c_ yield a $\chi _{{\mathrm{eff}}}^{(3)}$ that is about one order of magnitude larger (10^−6^ esu)^8,11^, but no conclusive physical explanation for the difference in *Z*-scan nonlinearities measured in the two lifetime/pulse-length scenarios was given in those works. Yet, when looking at our results for the ps-pumped graphene-covered SiO_2_ waveguides featuring *T*_0_ ≈ *τ*_c_, an effective graphene nonlinearity $\chi _{{\mathrm{eff}}}^{(3)} \propto - 10^{ - 6}$ esu would be needed to account for the observed strong broadening factors. This value is also one order of magnitude larger than the $\chi _{{\mathrm{eff}}}^{(3)}$ extracted in our previous work from *γ*_gr-on-Si_ where $\tau _{\mathrm{c}} \gg T_0$ applied. We point out that, in addition to nonlinearity magnitudes, our experiments have also revealed the nonlinearity behavior as a function of propagation distance in the hybrid waveguides, clearly indicating the occurrence of SPCR instead of conventional SPM. Such measurements as a function of interaction length are less straightforward to implement in *Z*-scan experiments with free-space light beams propagating across atomically thin graphene sheets. Finally, it is interesting to note that for *Z*-scan measurements with fs-scale pulse excitation graphene’s nonlinearity is expected to increase with increasing pulse length *T*_0_, as can be derived from our graphene-on-Si results with $K = - \sigma _{{\mathrm{FCR}}}\sqrt {\left( {\eta _{{\mathrm{1PA}}}\,T_0} \right)/\left( {\hbar \omega \,P_0} \right)}$ for $\tau _{\mathrm{c}} \gg T_0$.

## Discussion

In conclusion, we have revealed the origin of the long-standing quantitative discrepancy between the theoretical *χ*^(3)^-based predictions and the experimental observations for graphene’s nonlinear phase-modulation ability in SPM and *Z*-scan investigations. Rather than relying on graphene’s electronic *χ*^(3)^, the measured phase modulation effects arise from a more complex phenomenon we refer to as saturable photoexcited-carrier refraction. Depending on the ratio between the carrier lifetime and the pulse length used, the SPCR-induced physics can either mimic the tendencies of conventional *χ*^(3)^-based phase modulation, or produce a totally different behavior. A striking example of the latter is the exponentially growing spectral broadening we have seen for ps pulses propagating in graphene-covered SiO_2_-core waveguides. This behavior relies on both up-chirping and down-chirping along variable chirp profiles and is totally different from the earlier studied FCR-induced spectral broadening in 3D-semiconductor waveguides. The determining factors for this exceptional behavior are the short *τ*_c_ for photoexcited carriers in graphene and the saturability of the 2D material. This exponential broadening growth opens up new routes to exploiting graphene for on-chip spectral broadening of laser signals and, in case of an optimized balance between the one-photon absorption efficiency and the SPCR efficiency, for actually creating new wavelengths in on-chip frequency-comb and supercontinuum light sources. Furthermore, as the optical pulse power turns out to have a limited impact on the broadening efficiency, one could even envisage frequency-comb and supercontinuum generation at record-low input power levels. In other words, our findings thoroughly change how nonlinear-optical devices could be operated when enhanced with graphene. The SPCR model presented here provides a qualitative and quantitative description for both the cases where such extraordinary broadening behavior is produced and the cases where conventional *χ*^(3)^-based broadening is mimicked. It also reveals the physical origin of the negative sign of graphene’s effective $\chi _{{\mathrm{eff}}}^{(3)} = - 10^{ - 7}$ esu as extracted from our previous on-chip broadening experiments and from *Z*-scan experiments where mimicked *χ*^(3)^-based behavior was produced in the regime of $\tau _{\mathrm{c}} \gg T_0$. Although the focus in our work has been on graphene-based nonlinear phase modulation, it is interesting to note that also for other nonlinear processes such as four-wave mixing and third-harmonic generation effective nonlinearities of a similarly large magnitude have been measured. This calls for investigating also these other nonlinear processes in graphene^1–7^ and 2D materials in general^46–49^ by means of free-carrier dynamics modeling as we used here. We note that for some of those experiments (e.g., the third-harmonic generation measurements reported in ref. ^7^) nonlinearity magnitudes below 10^−7^ esu were reported, but also for those measurements the insights we have obtained here regarding, e.g., the impact of the pulse duration on the nonlinearity strength in *Z*-scan experiments can be relevant. A more in-depth understanding of the fundamental 2D-material nonlinearities will finally allow the full exploitation of their great potential in next-generation (on-chip) nonlinear-optical devices.

## Methods

### Fabrication of graphene-covered SiO_2_-core waveguides

Photonic waveguides with spot-size converters at the end facets were fabricated in a multi-project wafer run of the LioniX foundry (https://www.lionix-international.com). The spot-size converters allow edge coupling with flat-cleaved fibers placed at the waveguides’ input and output facets, yielding a fiber-chip-fiber coupling efficiency around −10 dB. The cross-sectional waveguide geometry consists of a SiO_2_ core region (thickness around 500 nm) with above and below a stripe of Si_3_N_4_ (stripe thickness around 170 nm). The foundry also provided a top oxide layer above the upper Si_3_N_4_ stripe (see Supplementary Note 1 and Supplementary Figure 1). The photonic chips delivered by the foundry were 8 mm wide, and featured a top oxide thickness of about 300 nm above the waveguides while above the spot-size converter regions the oxide thickness was several μm. The 300 nm-thick oxide above the waveguides was then almost completely removed using h-BF etching prior to the graphene transfer, so that the remaining top oxide thickness was below 50 nm. The waveguides used in the broadening experiments all feature a stripe width of 1300 nm, yielding single-mode quasi-TE operation in the telecom band around 1550 nm.

Monolayer graphene was grown on a high-purity 35 μm-thick polycrystalline copper foil using chemical vapor deposition (CVD) of methane in a commercially available Black Magic Pro system. The time and flow rate settings were optimized such that monolayer graphene growth was ensured. Afterwards a 200 nm-thin layer of poly(methyl methacrylate) (PMMA) was spincoated on top of the graphene. After electrochemical delamination of the CVD-grown graphene film in a 1 M aqueous solution of potassium chloride, it was deposited on top of the photonic chip along the wet-transfer method^28^ and annealed at 120 °C for 12 h. To create graphene sections of varying length on the waveguides, the graphene with the PMMA on top was patterned using O_2_ plasma etching and a mechanical mask laser-cut in a 200 μm-thick steel plate to define the openings to be etched^29^. The PMMA was not removed after the etching step, so that the refractive index in the region above the graphene remained sufficiently high for having a strong optical field in the monolayer. By experimentally comparing spectral broadening with and without PMMA, we have verified that the PMMA has no influence on the observed broadening behavior. We have also checked through on-chip Hall-effect measurements that the graphene exhibits a usual level of unintentional doping^28^ (see Supplementary Note 2 and Supplementary Figure 3).

### Optical experiments

The pulsed laser source used to excite the graphene-covered waveguides is an optical parametric oscillator OPO (APE Levante IR) generating picosecond pulses at a repetition rate of 80 MHz. To create input pulses with either a positive or negative chirp, we made use of the chirp modification that the OPO pulses experience when injected in sufficiently long fiber sections^14^. To determine the appropriate fiber length for achieving a given chirp modification, one can make use of our pulse-chirp control method based on the generalized-length formalism^50^. The chirped pulses were then coupled into and out of the hybrid waveguides by means of flat-cleaved fiber probes positioned horizontally at the waveguides’ end facets, and their peak power was varied using a variable optical attenuator. A 99:1 coupler was inserted in front of the incoupling fiber probe to split off 1% of the input pulse power towards a power meter (Newport 2832-C with 818-SL detector head) and an optical spectrum analyzer (Yokogawa AQ6370D) for monitoring both the input power and spectrum. Along the same approach the output power and spectrum were measured just behind the outcoupling fiber probe using a second power meter and optical spectrum analyzer. Prior to the optical measurements, the input pulse duration and phase/chirp profile were characterized by means of a frequency-resolved optical gating (FROG) instrument (Coherent Solutions HR150).

### Numerical simulations

The quasi-TE mode in the graphene-covered waveguide as shown in Fig. 1a was calculated using Lumerical MODE Solutions software. The pulse propagation formula Eq. (4) was solved by means of an Adams method, and the free-carrier rate Eq. (5) was solved along the same approach used for the free-carrier rate equation in silicon waveguides^32^.

### Data availability

The data that support the findings of this study are available from the corresponding author upon reasonable request.

## Electronic supplementary material

Supplementary Information
