# Supplementary material for: Graphene’s nonlinear-optical physics revealed through exponentially growing self-phase modulation
Source: Nat Commun. 2018 Jul 11;9:2675. doi: 10.1038/s41467-018-05081-z (PMC6041291; doi:10.1038/s41467-018-05081-z)
Supplement: Supplementary file 1 — Supplementary Information [file 41467_2018_5081_MOESM1_ESM.pdf]

Supplementary Information for:
Graphene's nonlinear-optical physics revealed through exponentially growing self-phase modulation

Nathalie Vermeulen *et al.*

Supplementary Note 1: Cross-sectional geometry of SiO_2-core waveguides

We employed waveguides fabricated in a multi-project wafer run of the LioniX foundry and with a cross-sectional waveguide geometry as shown in the scanning-electron microscope (SEM) picture of Supplementary Figure 1. The cross-section consists of a SiO_2 core region (thickness around 500 nm) contained between two horizontal Si_3N_4 stripes (stripe thickness around 170 nm) with customizable width, which is about 0.7 µm in the example of Supplementary Figure 1. This double-stripe geometry sustains quasi-TE polarized light with a substantial part of the modal power contained in the SiO_2 core (see Fig. 1(a)). The waveguides delivered by the foundry feature a top oxide layer above the upper Si_3N_4 stripe, but we removed this layer prior to graphene deposition (see Methods).

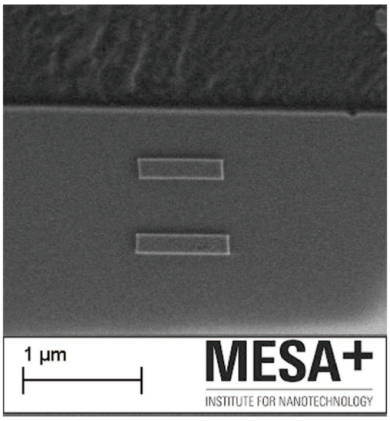

Supplementary Figure 1: Scanning-electron microscope image of the cross-sectional geometry of the waveguides as delivered by the LioniX foundry. In this example the Si_3N_4 strip width is about 0.7 µm. The image was made at an angle of 70 degrees with respect to the normal on the chip surface (image courtesy to LioniX International).

Supplementary Note 2: Raman and Hall-effect characterization of graphene

To characterize the material properties of the graphene grown by means of chemical vapor deposition (CVD), covered with poly(methyl methacrylate) (PMMA) and transferred on the SiO_2-core waveguides, we measured the Raman spectrum of the graphene on the photonic chip with a 532 nm laser. We ensured that the laser beam was properly focused on the graphene below the PMMA top layer, and measured a strong Raman signal from the 2D material showing its monolayer nature with sharp and well-defined features (Supplementary

Figure 2). The almost equal magnitude of the measured G and 2D peaks is due to annealing effects [1], as was also the case for the graphene used in our previous work [2, 3]. We also see that due to the PMMA cover layer there is a blue-shift in the peak positions [1, 4] as compared to those usually measured for graphene without PMMA on top (G and 2D peak positions typically around 1585 and 2680 cm^{-1}, respectively). As the graphene was annealed with the PMMA on top, the spectrum also shows features stemming from hydrocarbons [1].

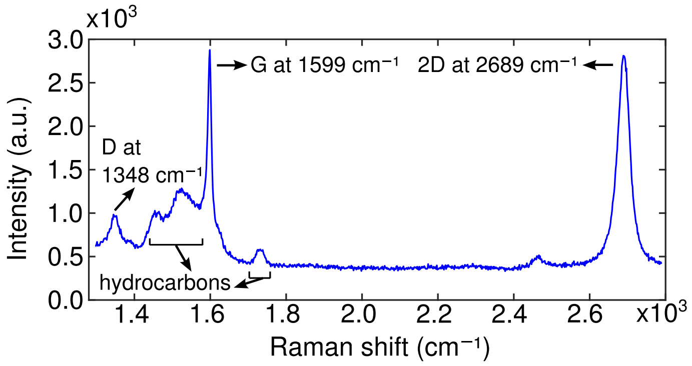

Supplementary Figure 2: Raman spectrum of the graphene top layer on the photonic chip with SiO_2-core waveguides. The graphene was covered with poly(methyl methacrylate) and the spectrum was measured with 1 mW 532 nm laser excitation.

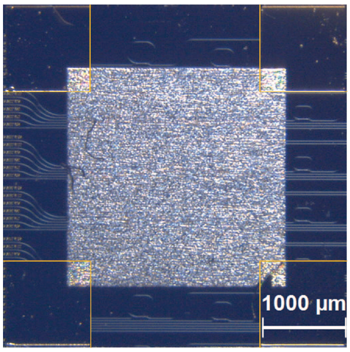

Supplementary Figure 3: Square oxygen-plasma-etched graphene section on top of the photonic chip and with four electrical contacts in the corners for Hall-effect measurements. The square graphene section was covered with poly(methyl methacrylate) and was situated in a sacrificial area of the chip containing waveguides that were not needed for the waveguide experiments in this work.

To determine graphene's charge carrier concentration n, charge carrier mobility μ and sheet resistance R, we carried out Hall-effect measurements in van der Pauw geometry on a square PMMA-covered oxygen-plasma-etched graphene section (size: 2.5 mm × 2.5 mm) directly on top of the photonic chip, as illustrated in Supplementary Figure 3. This yielded:

$n = +6.5 \times 10^{12}$ cm^{-2}; $\mu = 1312$ cm^2/(V s); $R = 732$ Ω/sq

The electrical parameters of the sample indicate that the CVD-grown graphene is of good quality and features usual unintentional p-doping [5].

Supplementary Note 3: Modeling results for conventional self-phase modulation

Figs. 2(a)-(b) clearly show that the modeling results for conventional self-phase modulation (SPM) in the graphene-covered SiO_2-core waveguides cannot quantitatively describe the experimentally observed broadening in these waveguides. The zoom-in depicted here in Supplementary Figures 4(a)-(b) demonstrates that neither the qualitative behavior predicted by conventional-SPM modeling matches with that of the experimental broadening. In contrast, our saturable photoexcited-carrier refraction (SPCR) theory adequately describes the experimental data both quantitatively and qualitatively.

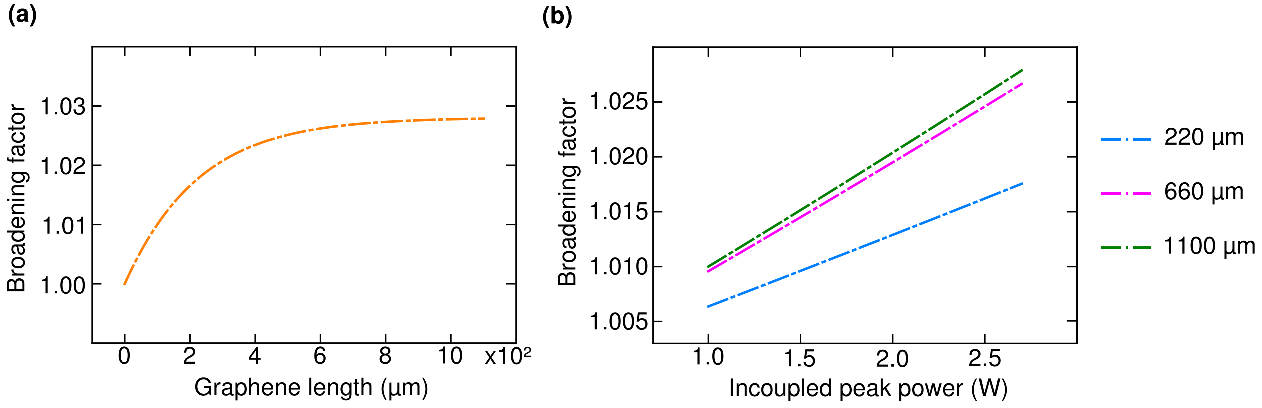

Supplementary Figure 4: Zoom-in of the modeling results for conventional self-phase modulation. Zoom-in of (a) the modeled broadening factor as a function of graphene length for an incoupled peak power of 2.7 W, and (b) the modeled broadening factor as a function of incoupled peak power for graphene lengths of 220 µm, 660 µm, and 1100 µm. These modeling results are generated using graphene's strong effective $\chi^{(3)}_{\mathrm{eff}} = -10^{-7}$ esu as extracted from earlier experiments.

Supplementary Note 4: Analytical solution for graphene-covered Si waveguides

For the graphene-covered semiconductor (namely Si) waveguides used in our earlier work [3], the SPCR physics taking place in the graphene can be modeled using Eqs. (4)-(5), with $N_{\mathrm{c}}^{\mathrm{gr+wg}}$ in Eq. (5) the average carrier density defined over the entire cross-section of the hybrid waveguides, with $N_{\mathrm{c}}^{\mathrm{gr}}$ in Eq. (4) equal to $\sqrt{N_{\mathrm{c}}^{\mathrm{gr+wg}}}$, and with $\eta_{\mathrm{1PA}} = \alpha_{\mathrm{eff}}/D$ where

D represents the waveguide thickness (220 nm in this case). The parameter values to be implemented are enlisted in Supplementary Table 1.

Supplementary Table 1: Values of the parameters in Eqs. (4)-(5) for graphene-covered Si waveguides.

	Value	Reference
α_{eff} (m^{-1})	30394	Transmission exp. [3]
τ_{c} (ps)	200	[6]
N_{sat} (m^{-2})	10^{17}	[7]
σ_{FCR} (-)	$1(\pm 0.2) \times 10^{-5}$	See data hybrid SiO_2-core waveguides

As shown in Fig. 5, Eqs. (4)-(5) modeling the free-carrier refraction effects only in the graphene top layer and not in the underlying Si waveguide already provide a close match with the experimental results, better than the conventional-SPM model. The time evolution of the optical pulse power, the graphene-generated free-carrier density, and the instantaneous frequency at different distances in the graphene-covered Si waveguides is shown in Supplementary Figure 5.

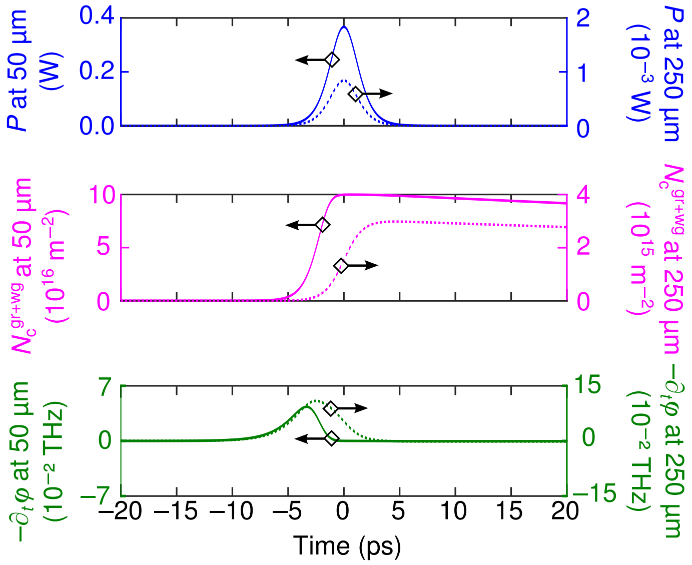

Supplementary Figure 5: Time evolution of the optical and carrier-related quantities at different distances in the graphene-covered Si waveguides. Time evolution of the optical power P (blue), the graphene-generated free-carrier density $N_{\mathrm{c}}^{\mathrm{gr+wg}}$ (magenta), and the instantaneous frequency $-\partial_t \varphi$ (green), as obtained numerically. The solid and dashed curves correspond to, respectively, 50 µm and 250 µm propagation distance.

Since the free-carrier lifetime τ_{c} is much longer here than the 3 ps excitation pulses, we have $\tau_{\mathrm{c}} \gg T_0$, which allows extracting from Eqs. (4)-(5) the following approximated

analytical solution for the SPCR-induced chirp in graphene-on-Si:

$$\partial_\tau \varphi(z,\tau) = \left[-\,\sigma_{\mathrm{FCR}} \sqrt{\frac{\eta_{\mathrm{1PA}}\, T_0}{\hbar\omega\, P_0}} \right] P_0 \, \frac{|\tilde{U}(\tau)|^2}{\sqrt{\int_{-\infty}^{\tau} |\tilde{U}(\tau')|^2 \mathrm{d}\tau'}} \big[\Theta_3 \, \mathrm{e}^{-\frac{1}{2}\alpha_{\mathrm{eff}} z} \, \alpha_{\mathrm{eff}}^{-1} \big] \big|_z^0$$

(Supplementary Equation 1)

with $\Theta_3 = \sqrt{[1-\mathrm{e}^{-x}]/x}$ where $x = (\eta_{\mathrm{1PA}}\, \mathrm{e}^{-\alpha_{\mathrm{eff}} z}\, P_0\, T_0 \int_{-\infty}^{\tau} |\tilde{U}(\tau')|^2 \mathrm{d}\tau')/(\hbar\omega N_{\mathrm{sat}})$. In Supplementary Equation 1 the quantity $-\sigma_{\mathrm{FCR}}\sqrt{(\eta_{\mathrm{1PA}}\, T_0)/(\hbar\omega\, P_0)}$ acts as proportionality constant K.

Supplementary Note 5: Sensitivity of SPCR model to changes in parameter values

To test the sensitivity of the SPCR modeling results for graphene-covered SiO_2-core waveguides to changes in the effective decay time τ_{c}, we carry out new numerical simulations for the broadening factor as a function of graphene length with this time $\tau_{\mathrm{c}} = 100$ fs instead of 1 ps as used in the main manuscript. Supplementary Figure 6 depicts the broadening factors for both $\tau_{\mathrm{c}} = 100$ fs and $\tau_{\mathrm{c}} = 1$ ps, and shows that the SPCR model has a rather modest sensitivity to this one order of magnitude change in the effective decay time. At the same time, when the effective decay time is increased by several orders of magnitude, we enter a totally different broadening regime as can be seen in the results for the graphene-covered Si waveguides. In other words, the model's sensitivity when switching from a decay time of 1 ps to a decay time of several hundreds of picoseconds should be interpreted as an adequate representation of the very different broadening behavior when switching to another type of graphene-covered waveguide with very different relaxation properties. We note that within the regime of large $\tau_{\mathrm{c}} \gg T_0$ as is the case for the graphene-covered Si waveguides, the modeling results become essentially insensitive to changes in τ_{c} (see the approximated analytical chirp expression of Supplementary Equation 1 which does not depend on the decay time).

Turning now to the model's sensitivity for e.g. the value of N_{sat}, Eq. (6) for the graphene-covered SiO_2-core waveguides indicates that changes in N_{sat} will have a larger impact on the modeling results than changes in τ_{c} as these two parameters take up different roles in Eq. (6). Indeed, while τ_{c} contributes to both the numerator and denominator, hence limiting the impact of variations in τ_{c}, N_{sat} is uniquely present in the denominator of Eq. (6). At the same time, we remark that the factor containing N_{sat} in Eq. (6) also contains τ_{c}, and

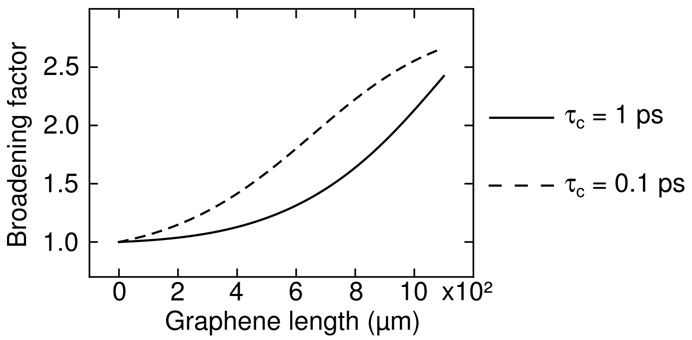

Supplementary Figure 6: Numerically simulated broadening factor for the graphene-covered SiO_2-core waveguides in case of two different decay times. In addition to $\tau_c = 1$ ps as used in the main manuscript, also $\tau_c = 100$ fs is considered here.

the factor in the numerator where τ_c occurs also contains σ_{FCR}. This implies that the same macroscopically observed broadening behavior can be modeled through different combinations of parameter values for τ_c, N_{sat} and σ_{FCR}. Hence, our model is robust against possible deviations of the actual parameter values in our graphene-covered SiO_2-core waveguides from the values used in Table 1.

Supplementary References

[1] Hong, J. *et al.* Origin of New Broad Raman D and G Peaks in Annealed Graphene. *Scient. Reports* **3**, 2700 (2013).

[2] Van Erps, J. *et al.* Laser ablation- and plasma etching-based patterning of graphene on silicon-on-insulator waveguides. *Opt. Express* **23**, 26639–26650 (2015).

[3] Vermeulen, N. *et al.* Negative Kerr Nonlinearity of Graphene as seen via Chirped-Pulse-Pumped Self-Phase Modulation. *Phys. Rev. Appl.* **6**, 044006 (2016).

[4] Sobon, G. *et al.* A tunable, linearly polarized Er-fiber laser mode-locked by graphene/PMMA composite. *Laser Phys.* **23**,125101 (2013).

[5] Ciuk, T. *et al.* Properties of chemical vapor deposition graphene transferred by high-speed electrochemical delamination. *J. Phys. Chem. C* **117**, 20833–20837 (2013).

[6] Gu, T. *et al.* Regenerative oscillation and four-wave mixing in graphene optoelectronics. *Nature Photon.* **6**, 554–559 (2012).

[7] Bao, Q. *et al.* Atomic-Layer Graphene as a Saturable Absorber for Ultrafast Pulsed Lasers. *Adv. Funct. Mater.* **19**, 3077–3083 (2009).
